# Red Blood Cell Responses during a Long-Standing Load in a Microfluidic Constriction

**DOI:** 10.3390/mi8040100

**Published:** 2017-03-26

**Authors:** Mitsuhiro Horade, Chia-Hung Dylan Tsai, Hiroaki Ito, Makoto Kaneko

**Affiliations:** Department of Mechanical Engineering, Osaka University, Suita 565-0871, Japan; horade@mech.eng.osaka-u.ac.jp (M.H.); ito@hh.mech.eng.osaka-u.ac.jp (H.I.); mk@mech.eng.osaka-u.ac.jp (M.K.)

**Keywords:** microfluidics, long-standing load, cell manipulation, red blood cell

## Abstract

Red blood cell responses during a long-standing load were experimentally investigated. With a high-speed camera and a high-speed actuator, we were able to manipulate cells staying inside a microfluidic constriction, and each cell was compressed due to the geometric constraints. During the load inside the constriction, the color of the cells was found to gradually darken, while the cell lengths became shorter and shorter. According to the analysis results of a 5 min load, the average increase of the cell darkness was 60.9 in 8-bit color resolution, and the average shrinkage of the cell length was 15% of the initial length. The same tendency was consistently observed from cell to cell. A correlation between the changes of the color and the length were established based on the experimental results. The changes are believed partially due to the viscoelastic properties of the cells that the cells’ configurations change with time for adapting to the confined space inside the constriction.

## 1. Introduction

There are established relationships between the deformability of red blood cells (RBCs) and human diseases, such as Malaria, Spherocytosis and Cryohydrocytosis [1,2,3,4,5]. While many works have focused on RBC deformability through short-duration compressions, such as transit time through a constriction or micro-slit [6,7,8,9,10,11], few works have focused on RBC characteristics after long-standing loads [12,13]. RBC responses under a long-standing load reflect how a RBC changes while being plugged in microcirculation. For example, Baez et al. found different flow characteristics of oxygenated and partially deoxygenated red cells plugging in microcirculation [14]. Therefore, this work is motivated by the idea of investigating cell responses during a long-standing load using microfluidic constriction.

**Figure 1:**
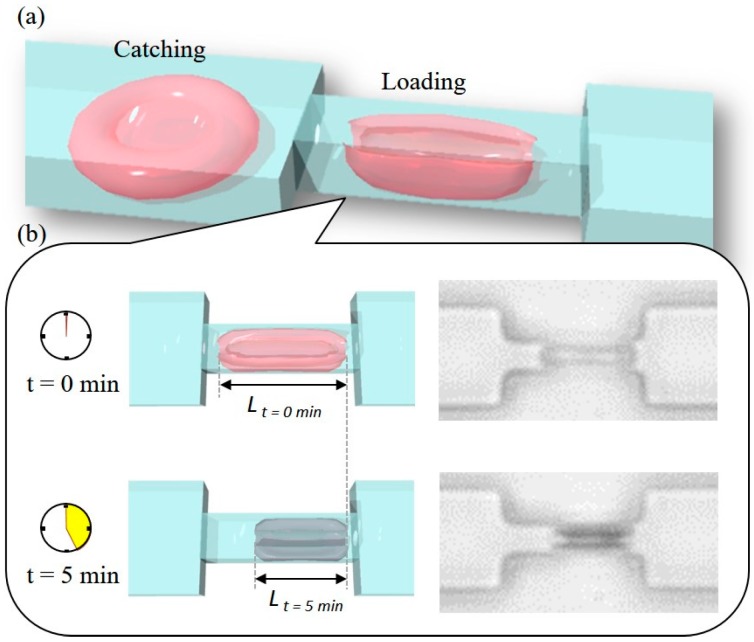
An overview of the proposed method and example results. (**a**) An illustration of cell manipulation for the long-standing load; (**b**) illustrations and experimental images of a red blood cell (RBC) before and after a 5 min load.

Figure 1 shows an overview of the proposed idea and sample RBC photos at the beginning and the end of a long-standing load. A constriction channel is placed in a microfluidic device as illustrated in Figure 1a. There are two phases of the experiment. First, a target RBC is positioned in front of the narrow channel for recovering from prior deformation as the “Catching” phase. Afterward, the RBC is moved into the constriction channel for compression as the “Loading” phase. The cell manipulations are performed by a vision-feedback system where the real-time cell position is computed for proper actuator output for moving the cell to a target position. The RBC is maintained inside the constriction for a specified duration as the long-standing load, and a camera is set for observing the responses of the cell during the load.

Figure 1b illustrates a RBC before and after the 5 min load with two sample images from the experiments. At the beginning of the load, the RBC is slender and almost transparent. After the 5 min load, the color of the RBC is darkened, and the horizontal length is significantly shortened.

A time-lapse sample video can be found in the Supplementary Materials Video S1. Quantitative evaluations of the amount of change in cell color and cell length are measured and presented in this paper. The correlation and interpretation from the mechanical aspect are discussed based on the experimental results.

In this paper, we particularly focus on the evolution of the RBC color and length during a long-standing load. These newly found cell properties could provide new insights or a different evaluation index for cell evaluation.

## 2. Related Works

RBC deformability has been found to be correlated with certain diseases [5,15,16,17,18]. For example, RBCs with reduced deformability are found in obese patients and in those with diabetic foot diseases [15,16]. Malaria is a well-known blood-related disease that reduces RBC deformability [17,18]. Unusual morphology of RBCs has been found from those of the compound heterozygous for hemoglobin S and hemoglobin C [19]. There are different approaches for investigating RBC deformability [20,21]. For example, Tözeren et al. used micropipette for developing a constitutive equation for the RBC membrane [22]. Brandao et al. employed optical tweezers for measuring RBC elasticity [23]. Dulinska et al. applied an atomic force microscope (AFM) for measuring the stiffness of erythrocytes [24]. Among different evaluation methods, microfluidic constrictions have become popular in the last decade because of their precise fabrication and accurate control [25,26,27]. For example, Zheng et al. put RBCs through a constriction for high-throughput biophysical measurements [28]. Sakuma et al. evaluated the cell fatigue state by inducing continuous and repetitive deformation [29]. There are also works investigating RBC responses after long-standing loads. For example, Fischer discovered the shape memory of RBCs based on their recovery from the deformation by shear stress up to 4 h [12]. Markle et al. investigated the viscoelastic properties of the RBC membrane by characterizing their shape after constant-pressure aspiration up to 90 min [13]. Through the microfluidic platform, it becomes possible to stably monitor cells’ behaviors and responses during a fixed-displacement load.

In our previous works, we succeeded in applying long-standing loads to RBCs by high-speed cell manipulation and discussed how the RBCs recover after the load [30,31]. Preliminary results of the RBC color and length changes during the load were independently observed and presented at recent conferences [32,33]. In this work, we firstly present a comprehensive analysis of cell changes during the long-standing load. Furthermore, a correlation between the changes of the RBC color and length is found according to the experimental results.

## 3. Experimental System and Procedure

### 3.1. Cell Manipulation System

Cell manipulation is the key technique for this work. Without an accurate cell manipulation for keeping RBCs inside the constriction, RBC response under long-standing load cannot be properly observed. Micropipettes or robotic microgrippers may also apply a constant load to RBCs, but they usually take much longer time to locate a cell [12,13,21,34]. Thus, the microfluidic system offers a more convenient platform for the test.

**Figure 2:**
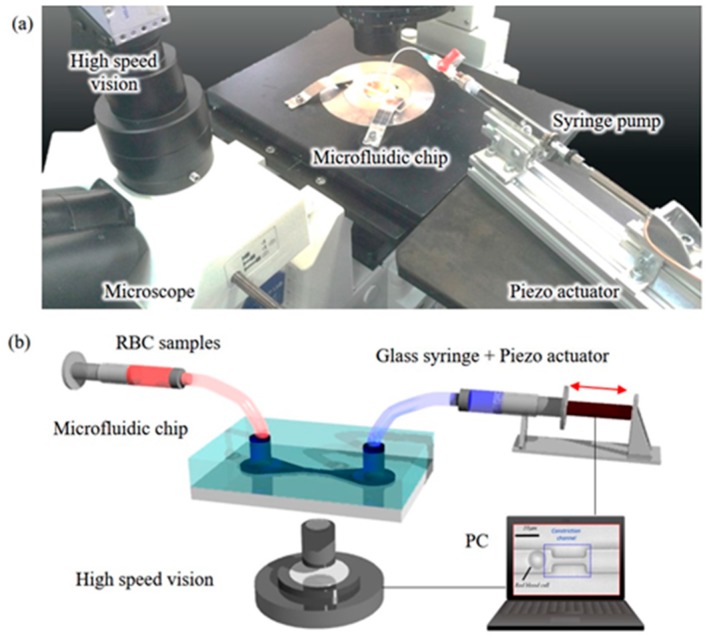
The experimental setup. (**a**) A photo of the setup; (**b**) a schematic view of the experimental system. The RBC is manipulated by a vision-feedback control system with real-time RBC position and microfluidic flow control.

Figure 2 shows the experimental setup of cell manipulation system, which is composed of a poly-dimethylsiloxane (PDMS) chip, a piezoelectric actuator (MESS-TEK: M-2655s(c), MESS-TEK Ltd., Saitama, Japan), a syringe (SGE Analytical Science: 2.5MDF-LL-GT, LGE Japan Ltd., Osaka, Japan), a high-speed camera (Photoron: IDP-Express R2000, Photron Ltd., Tokyo, Japan), a microscope (OLYMPUS: IX71, Olympus Co., Tokyo, Japan), and a personal computer (PC). The high-speed camera and high-speed actuator are two main components for the cell manipulation inside a microfluidic device. The camera provides real-time cell position with the frame rate of 1000 frames per second (fps) while the high-speed piezoelectric actuator can rapidly adjust cell position by generating appropriate fluid flow inside the microfluidic chip. An animation demonstrating the manipulation concept can be found in Supplementary Materials Video S2. The captured image frames are processed by image methods for determining the cell position, and the position is sent to a proportional-integral-derivative (PID) controller for computing a suitable output signal to move the actuator. The algorithms are implemented in a C program. The sampling rate in the program is 1000 Hz, which means a slight change of cell position would be immediately compensated by the system within one millisecond [35].

### 3.2. Design and Fabrication of PDMS Chip

**Figure 3:**
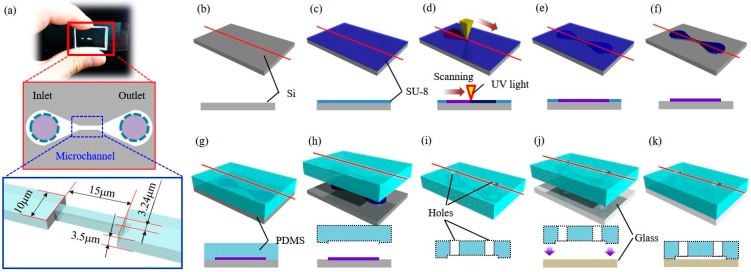
Design and fabrication of the microfluidic chip. (**a**) Schematic view of the microfluidic chip. The device consists of an inlet, an outlet, a microchannel (3.5 µm in height and 10.0 µm in width), and narrow channel (3.5 µm in height, 15.0 µm in length and 3.24 µm in width); (**b**–**f**) the step-by-step photolithography for mold fabrication; (**g**–**k**) the step-by-step procedure for poly-dimethylsiloxane (PDMS) chip fabrication from the mold.

Figure 3 illustrates the fabrication of the microfluidic chip used in this work. Figure 3a shows the dimensions of the channel design. The widths of the main channel and the constriction are 10.0 and 3.24 µm, respectively. The channel height is 3.5 µm for the both parts. The dimensions of the channel are designed for introducing deformation to the tested RBCs. Although RBCs are known having extraordinary deformability and can go through 1–3 µm pores without lysis, the 3.24 µm wide channel is designed to let the tested RBCs deform around 50% while the length of 15 µm is to make sure all deformed RBCs are kept inside the constriction. RBC deformation through the different width of the channel has been previously investigated [36]. An inlet and an outlet are on the two sides of the main channel for RBCs feeding and the syringe pump connection. Figure 3b–f and Figure 3g–k illustrate the fabrication of the mold and microfluidic chip, respectively. In the mold fabrication, a silicon substrate is spin coated with a layer of photoresist (SU-8 3005, Microchem Corp., Newton, MA, USA) before being exposed to ultraviolet (UV) light for lithography. The mold is developed using propylene glycol monomethylether acetate (PM) thinner and isopropyl alcohol (IPA). The mixture of PDMS and curing agent (Sylgard^®^ 184, Dow Corning Corp., Midland, MI, USA) are poured over the fabricated mold in a container for molding. After the PDMS is cured, the PDMS chip is peeled off from the mold and two holes are punched for the inlet and outlet. Finally, the microfluidic chip is bonded to a slide glass using a plasma device (Cute-MP, Femto Science Inc., Gyeonggi-Do, Korea).

### 3.3. RBC Sample Preparation and Control Sequence

Blood for the experiment was provided by a volunteer subject who has read and agreed with the consent of the test. About 5 µL of blood was taken by a sterilized lancet from a fingertip of the subject. The blood is diluted 100 times by standard saline for reducing the number density of the RBCs in the test sample. The single cell test cannot be performed without the dilution due to the high density of RBCs in the whole blood. The sample solution is injected into the channel from the chip inlet, and then the syringe pump is connected to the outlet for the RBC manipulation. Because the changes of RBC color and length are faster at the beginning and gradually approaching to asymptotic values, the image sampling rates are set every 0.1, 1 and 10 s for the intervals of *t* = 0–1 s, *t* = 1–20 s and *t* = 20–290 s, respectively.

## 4. Experimental Results

**Figure 4:**
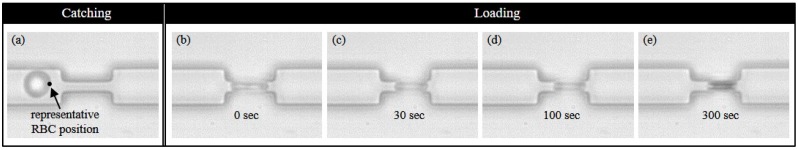
An example of a RBC in the microfluidic channel before and during loading. (**a**) The RBC at the catching phase; (**b**–**e**) The RBC response to the long-standing load at 0, 30, 100, and 300 s, respectively. The RBC’s color and length were changed with respect to the loading time.

Figure 4 shows an example RBC in the test. The RBC was firstly caught on the left of the constriction from a constant pressure-driven flow, as shown in Figure 4a. During the catching phase, the RBC was held steady for 2 s to recover from prior deformations, such as deformation caused by the shear stress from the flow. Afterward, a new target position was given as the right end of the constriction, and as a result, the RBC was moved into the constriction and was compressed. Figure 4b–e are the images of the RBC staying inside the channel for 0, 30, 100, and 300 s, respectively. In this example, we can directly observe the changes of the RBC color and length during the loading phase. The length of the RBC became shorter, while the color got darker. A time-lapse sample video can be found in Supplementary Materials Video S1.

**Figure 5:**
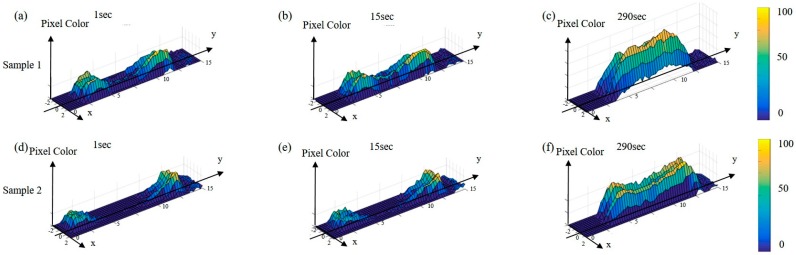
The color distributions of two different RBC samples at the loading times of *t* = 1 s, *t* = 15 s, and *t* = 290 s. (**a**–**c**) RBC sample 1 at the loading times. (**d**–**f**) RBC sample 2 at the loading times.

Figure 5a–c and Figure 5d–f show the color distribution of two different RBCs during the same long-standing load. The *x*, *y* and *z* axes in the figure represent the *x*, *y* positions, and the corresponding color value, respectively. The color is in standard 8-bit grayscale, where the color of each pixel ranges from 0 (white) to 255 (black). The durations of loading shown in Figure 5a,d, Figure 5b,e and Figure 5c,f are 1, 15 and 290, respectively. One consistent tendency for the two different RBCs, as well as all the tested RBCs, was that the overall pixel color of the cells got darker with respect to the loading time. However, the color patterns were different. The pattern around the center of the RBC in Figure 5c is flat whereas the one in Figure 5f is like a valley where the color of the central pixels is lighter than the surrounding pixels. The shrinkages of the RBC lengths during the load can also be observed in Figure 5. The length changes of RBC Sample 1 and 2 can be seen in Figure 5a–c and Figure 5d–f, respectively. The shrinkages of the RBCs on the top and bottom of Figure 5 were about 5% and 14% with respect to their initial lengths at *t* = 0 s. According to the two samples shown in Figure 5, we find that while different RBCs share a similar tendency of getting darker and shorter, they also have different responses in terms of the color patterns and the amount of shrinkage during the long-standing load.

**Figure 6:**
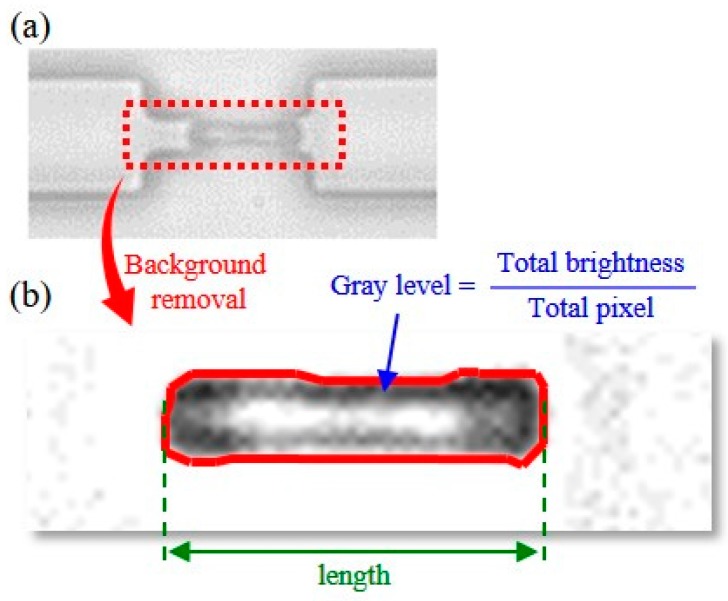
Determination of RBC gray level and length from a captured image. (**a**) A raw image frame; (**b**) the enhanced image by removing the background.

Figure 6 explains the image processing method for the representative cell color and length. Figure 6a shows a captured image from the camera. A background image was subtracted from each captured image for background removal. Figure 6b shows the cell image after background removal. Although the color on a RBC is not uniform and may have different patterns, such as the two examples shown in Figure 5, for the convenience of analysis, the gray level was defined as a representative color value for a RBC and was the average of the color values over the RBC. The RBC length is the distance between the left-most and right-most points of the detected cell area image as the green arrow indicates in Figure 6.

**Figure 7:**
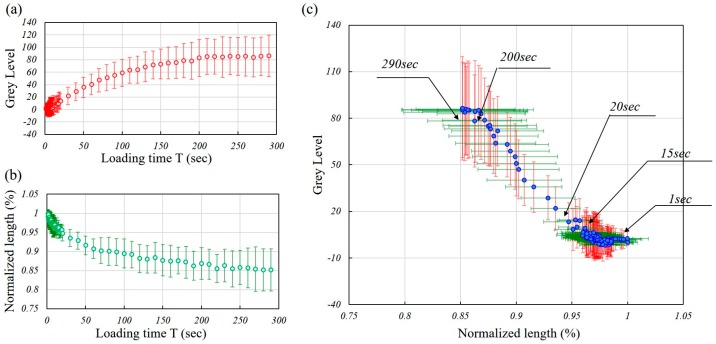
The experimental results of the gray level and cell length of the RBCs with respect to the loading time. (**a**) The change of the gray level with respect to the loading time; (**b**) the change of the cell length with respect to the loading time; (**c**) the change of the gray level with respect to the cell length at different instances.

Figure 7a,b show the change of gray levels and lengths of all the tested RBCs with respect to the elapsed time, while Figure 7c shows the correlation between the gray levels and lengths at different instances. Because different RBCs have different initial gray levels, the gray level at *t* = 0 s was normalized to 0 for the convenience of comparison. The data points and the error bar represent the averages and standard deviations of all the tested RBCs. The results in Figure 7a show that the gray level is increasing with respect to the elapsed time of loading. The curves of the gray level show an asymptotic approach to converging values for the RBCs.

The normalized cell length in Figure 7b was calculated as the ratio between every measured cell length and its initial length. The normalization was done for the convenience of comparing RBCs with different initial lengths in the narrow channel. The curves of the length were also logarithmically converging in the 5 min load. Figure 7c indicates a series of data sets at different elapsed times between the gray level and length. Negative correlations between the gray level and cell length are observed. An interesting observation from Figure 7c is that during the first 15 s of the loading, the cell length continuously decreased while the gray level remained almost constant. From 15 s to the end of the loading, the gray level increased, corresponding to the decrease of the cell length. That means the RBCs were shrinking first before changing color in the first 15 s of the loading.

## 5. Discussion

**Figure 8:**
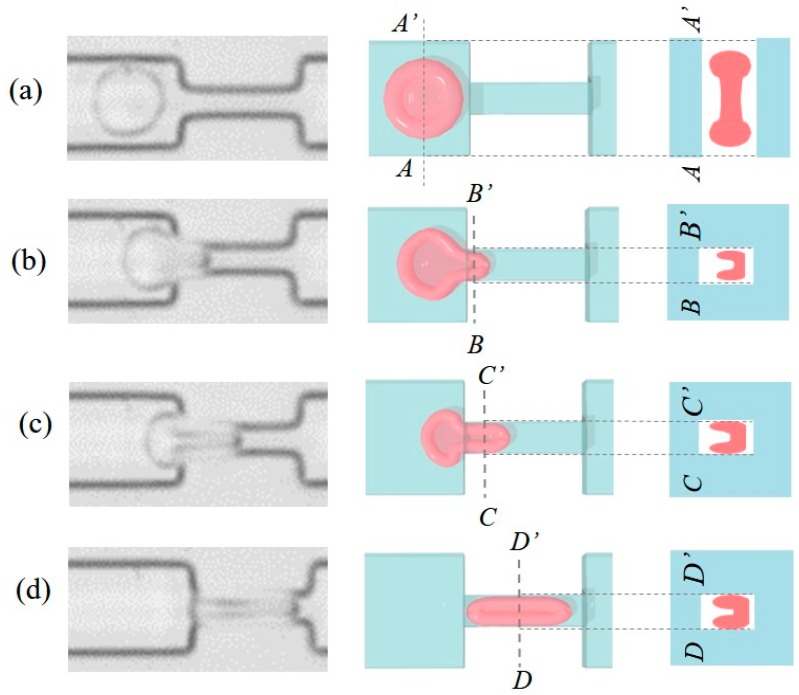
Interpretations of RBC deformation while squeezing into a constriction channel. (**a**) RBC before the deformation; (**b**) the tip of the RBC is compressed and folded for squeezing into the channel; (**c**) a greater area of the cross-section is filled by the RBC as it moves into the channel; (**d**) the RBC is fully squeezed into the narrow channel.

According to the experimental results in Figure 7, there is a correlation between the change of the gray level and the cell length during the long-standing loads. The decrease of the cell length happened earlier than the increase of the gray level. This fact says that the increase of the gray level may be brought about by the decrease of the cell length. For interpreting the phenomenon, we separately consider the cell behaviors in the entrance phase and the loading phase as follows:

Figure 8a–d show a RBC entering a constriction. The left column of Figure 8 shows a series of actual photos indicating the cell behavior during the entrance phase. A video clip of a RBC entering and exiting the constriction can be found in Supplementary Materials Video S3. A RBC is usually assumed being able to deform freely as long as the total surface area remains constant [37]. Based on the assumption, the possible shape changes of the RBC during the entrance phase are illustrated in the middle and right columns of Figure 8, which are the top view and the cross-sectional view, respectively. According to the photos, the RBC seems not only to be compressed but also folded in half for entering into the constriction. The cross-sectional area of the channel is gradually filled with the deformed RBC during the entrance. However, there are still vacancies around the deformed RBCs after the full entrance of the RBC.

**Figure 9:**
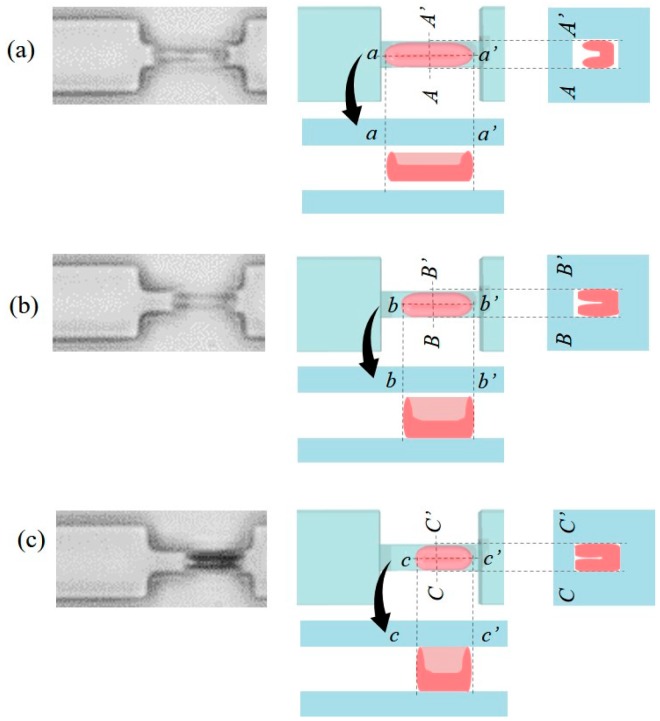
Interpretations of RBC changes during the long-standing load in the constriction. (**a**) Loading time *t* = 60 s; (**b**) loading time *t* = 180 s; (**c**) loading time *t* = 300 s.

Figure 9 illustrates a possible shape change of the RBC during the long-duration load. Illustrations of another cross-sectional view from the bottom are additionally included in Figure 9. The decreases of the cell length may happen as the cell gradually adapts to the confined space inside the constriction, and tries to fill the vacancies. This kind of shape adaption is generally known as strain creep for typical viscoelastic materials, and it means that the deformation of a compressed RBC is a function of time when the load or constraint is fixed. Based on the assumption of the constant surface area, this shape change makes the cell length shorten in the narrow channel. Furthermore, the adaption results in an equivalently thicker RBC from the top view as shown on the right of Figure 9c. The increase of the RBC thickness would reduce the transparency of the cell, and as a result, the cell is darkened and the gray levels increase as the experimental results show in Figure 7. However, further fluorescence or confocal microscopic images are needed to confirm the morphology changes of RBCs during the long-standing load and are the future direction of this work.

Figure 8 and Figure 9 provide a possible interpretation for the correlation between the changes of the RBC color and length from the perspectives of mechanical deformation. We would like to emphasize that the interpretation does not exclude other possible changes inside the RBC, such as reformation of the cytoskeleton or the exhaust of adenosine triphosphate inside the RBCs. In fact, it is very possible that there were fundamental changes of the RBCs based on the findings from the previous study on cell fatigue [24]. The other possible effects of RBCs under long-standing loads are worth investigating further from different perspectives, such as chemistry and biology, for RBC deformability.

## 6. Conclusions

The responses of RBCs during a long-standing load in a microfluidic constriction are experimentally investigated. The color of RBCs in the constriction becomes darker while the length of the RBCs shrinks with respect to the loading time. The spatial and temporal variations of the color change are analyzed. The results show both logarithmic changes of the color and the length of the RBCs. We also find an unanticipated negative correlation between the color and the RBC length according to the experimental results. Interpretation from the perspective of mechanical deformation is discussed. RBC responses under such a long-standing load are similar to a RBC being plugged in microcirculation inside a body, and the results could provide new insights into the time-dependent quality of plugged RBCs.

## Supplementary Materials

The following are available online at www.mdpi.com/2072-666X/8/4/100/s1; Video S1: A time-lapse video of the cell response during a long-standing load; Video S2: The conceptual animation of cell manipulation; Video S3: A slow-motion video of a RBC passing through a constriction channel showing the dynamic response of cell folding and unfolding.
